# O Índice Prognóstico Nutricional Oferece Novos Insights sobre a Circulação Colateral Coronariana na Angina Estável?

**DOI:** 10.36660/abc.20240144

**Published:** 2024-04-09

**Authors:** Ömer Faruk Çiçek

**Affiliations:** 1 Selcuk University Faculty of Medicine Department of Cardiovascular Surgery Konya Turquia Selcuk University, Faculty of Medicine, Department of Cardiovascular Surgery, Konya – Turquia

**Keywords:** Prognóstico Nutricional, Angina Estável, Circulação Colateral

A circulação colateral coronária (CCC) desempenha um papel crucial na manutenção do suprimento sanguíneo miocárdico, particularmente em pacientes com oclusão total crônica (CTO) e síndrome coronariana estável (SCE). Pesquisas recentes investigaram a associação entre marcadores inflamatórios e o desenvolvimento de CCC. No entanto, um novo estudo lança luz sobre uma perspectiva diferente, explorando a correlação entre o Índice Prognóstico Nutricional (IPN) e a formação de CCC em pacientes com SCE com CTO. O estudo, conduzido por Esenboga et al.,^1^ envolveu 400 pacientes com SCE com CTO, com o objetivo de investigar a potencial ligação entre IPN e CCC. Os resultados revelaram uma associação significativa entre níveis mais baixos de IPN e CCC pouco desenvolvido, independente de outros fatores de risco. Isto sugere que o IPN poderia servir como um biomarcador valioso para avaliar o CCC em pacientes com SCE.

A importância da CCC reside na sua capacidade de mitigar a isquemia miocárdica, aliviar os sintomas anginosos e melhorar o prognóstico do paciente. Embora estudos anteriores tenham explorado vários fatores que influenciam o desenvolvimento do CCC, como idade, comorbidades e marcadores inflamatórios, este estudo é pioneiro no exame do IPN como preditor. O IPN, calculado a partir dos níveis séricos de albumina e da contagem total de linfócitos, reflete o estado inflamatório e o estado nutricional de um indivíduo. Valores mais baixos de IPN significam aumento da carga inflamatória e pior estado nutricional, o que pode impedir a formação de CCC. O estudo propõe mecanismos plausíveis que ligam o baixo IPN ao mau CCC, envolvendo disfunção endotelial, diminuição da produção de óxido nítrico e comprometimento das respostas neovasculares.

Apesar dos seus insights inovadores, o estudo reconhece certas limitações, incluindo a sua natureza retrospectiva, o pequeno tamanho da amostra e a incapacidade de estabelecer causalidade. A ênfase do estudo no IPN como um preditor independente da CCC levanta questões sobre a causalidade e os mecanismos subjacentes. Embora os mecanismos propostos que ligam o baixo IPN à baixa CCC, como a disfunção endotelial e a diminuição da produção de óxido nítrico, sejam plausíveis, o estudo não fornece insights mecanicistas ou evidências experimentais para apoiar essas afirmações. Além disso, a natureza transversal do estudo impede o estabelecimento de relações temporais entre os níveis de IPN e o desenvolvimento do CCC, destacando a necessidade de investigações longitudinais para elucidar as vias causais.

Porém, ao revisar os critérios de exclusão e inclusão do estudo, nota-se que pacientes com histórico de infarto do miocárdio não foram incluídos no estudo. Contudo, considerando a baixa fração de ejeção observada em ambos os grupos, considera-se que o infarto do miocárdio silencioso não pode ser descartado em nenhum dos grupos. A presença ou ausência de tecido viável pode impactar significativamente o desenvolvimento colateral coronário, necessitando assim da padronização desta condição em ambos os grupos para uma análise comparativa precisa. Embora a análise retrospectiva restrinja inerentemente a capacidade de determinar este fator, continua a ser plausível considerar esta limitação no âmbito do estudo. A natureza retrospectiva da investigação limita inerentemente a disponibilidade de informações clínicas detalhadas sobre a presença ou ausência de tecido viável no miocárdio dos pacientes incluídos. Além disso, o estudo é limitado na sua capacidade de avaliar definitivamente o impacto do tecido viável no desenvolvimento colateral. Além disso, o reconhecimento desta limitação destaca a importância da cautela na interpretação dos resultados do estudo e sublinha a necessidade de futuras pesquisas abordarem esta lacuna de conhecimento.

Apesar da exclusão dos pacientes com frações de ejeção inferiores a 35% da coorte de estudo, é pertinente destacar que ambos os grupos de estudo manifestaram um nível moderado de comprometimento da função sistólica. Esta observação sublinha uma redução discernível na função ventricular esquerda, indicativa de desempenho cardíaco comprometido em toda a população amostrada. Contudo, à luz da reconhecida importância do tecido miocárdico viável na facilitação do desenvolvimento de colaterais coronárias, a relação precisa entre a disfunção sistólica observada e a potencial perda de tecido viável nos dois grupos permanece inadequadamente elucidada.^2 , 3^ Consequentemente, a incerteza inerente que rodeia esta associação introduz um elemento notável de ambiguidade na interpretação dos resultados do estudo, implicando assim o potencial de viés. No contexto de estudos retrospectivos, a eliminação abrangente de fatores que poderiam potencialmente introduzir preconceitos coloca desafios inerentes. Portanto, abordar possíveis variáveis de confusão requer uma consideração cuidadosa. Uma abordagem para este estudo poderia ter envolvido a inclusão específica apenas de pacientes com função sistólica normal, minimizando assim a influência do comprometimento do desempenho cardíaco nos resultados do estudo. Alternativamente, a confirmação da presença de tecido viável através de modalidades de imagem avançadas poderia ter oferecido outro caminho para mitigar potenciais vieses. Ao empregar essas estratégias, os pesquisadores poderiam ter aumentado a robustez e a confiabilidade de suas descobertas em desenhos de estudos retrospectivos.
