# Supplementary material for: O Índice Prognóstico Nutricional Oferece Novos Insights sobre a Circulação Colateral Coronariana na Angina Estável?
Source: Arq Bras Cardiol. 2024 Apr 9;121(2):e20240144. [Article in Portuguese] doi: 10.36660/abc.20240144 (PMC11081100; doi:10.36660/abc.20240144)
Supplement: Translation

# Translation: Does the Prognostic Nutritional Index Offer New Insights into Coronary Collateral Circulation in Stable Angina?

**Authors:** Ömer Faruk Çiçek

**Affiliations:** 1 Konya Turkey Selcuk University, Faculty of Medicine, Department of Cardiovascular Surgery, Konya – Turkey

**Keywords:** Prognostic Nutritional, Index Coronary, Collateral Circulation

Coronary collateral circulation (CCC) plays a crucial role in maintaining myocardial blood supply, particularly in patients with chronic total occlusion (CTO) and stable coronary syndrome (SCS). Recent research has delved into the association between inflammatory markers and CCC development. However, a novel study sheds light on a different perspective by exploring the correlation between the prognostic nutritional index (PNI) and CCC formation in SCS patients with CTO. The study, conducted by Esenboga et al.,^1^involved 400 SCS patients with CTO, aiming to investigate the potential link between PNI and CCC. The findings revealed a significant association between lower PNI levels and poorly developed CCC, independent of other risk factors. This suggests that PNI could serve as a valuable biomarker for assessing CCC in SCS patients.

The significance of CCC lies in its ability to mitigate myocardial ischemia, alleviate anginal symptoms, and improve patient prognosis. While previous studies have explored various factors influencing CCC development, such as age, comorbidities, and inflammatory markers, this study pioneers the examination of PNI as a predictor. The PNI, calculated from serum albumin levels and total lymphocyte count, reflects the inflammatory status and nutritional status of an individual. Lower PNI values signify increased inflammatory burden and poorer nutritional status, which may impede CCC formation. The study proposes plausible mechanisms linking low PNI to poor CCC, involving endothelial dysfunction, decreased nitric oxide production, and impaired neovascular responses.

Despite its groundbreaking insights, the study acknowledges certain limitations, including its retrospective nature, small sample size, and the inability to establish causality. The study's emphasis on PNI as an independent predictor of CCC raises questions about causality and underlying mechanisms. While the proposed mechanisms linking low PNI to poor CCC, such as endothelial dysfunction and decreased nitric oxide production, are plausible, the study does not provide mechanistic insights or experimental evidence to support these claims. Additionally, the cross-sectional nature of the study precludes establishing temporal relationships between PNI levels and CCC development, highlighting the need for longitudinal investigations to elucidate causal pathways.

However, upon reviewing the exclusion and inclusion criteria of the study, it is noted that patients with a history of myocardial infarction were not included in the study. Nevertheless, considering the observed low ejection fraction in both groups, it is considered that silent myocardial infarction cannot be ruled out in either group. The presence or absence of viable tissue can significantly impact coronary collateral development, thereby necessitating the standardization of this condition across both groups for accurate comparative analysis. Although retrospective analysis inherently restricts the ability to ascertain this factor, it remains plausible to consider this limitation within the scope of the study. The retrospective nature of the investigation inherently limits the availability of detailed clinical information regarding the presence or absence of viable tissue in the myocardium of the included patients. Additionally, the study is constrained in its ability to definitively assess the impact of viable tissue on collateral development. Furthermore, acknowledging this limitation highlights the importance of caution when interpreting the study's findings and underscores the necessity for future research to address this knowledge gap.

Despite the exclusion of patients with ejection fractions below 35% from the study cohort, it is pertinent to highlight that both study groups manifested a moderate level of impairment in systolic function. This observation underscores a discernible reduction in left ventricular function, indicative of compromised cardiac performance across the sampled population. However, in light of the acknowledged importance of myocardial viable tissue in facilitating the development of coronary collaterals, the precise relationship between the observed systolic dysfunction and potential viable tissue loss in the two groups remains inadequately elucidated.^2 , 3^Consequently, the inherent uncertainty surrounding this association introduces a notable element of ambiguity in the interpretation of the study findings, thereby implicating the potential for bias. In the context of retrospective studies, the comprehensive elimination of factors that could potentially introduce bias poses inherent challenges. Therefore, addressing potential confounding variables requires careful consideration. One approach for this study could have involved the specific inclusion of patients with normal systolic function solely, thus minimizing the influence of impaired cardiac performance on study outcomes. Alternatively, the confirmation of viable tissue presence through advanced imaging modalities could have offered another avenue for mitigating potential biases. By employing these strategies, researchers could have enhanced the robustness and reliability of their findings in retrospective study designs.
